# An Information Directory App InHouse Call for Streamlining Communication to Optimize Efficiency and Patient Care in a Hospital: Pilot Mixed Methods Design and Utility Study

**DOI:** 10.2196/23833

**Published:** 2022-01-27

**Authors:** George Schilling, Leonardo Villarosa

**Affiliations:** 1 Vidant Medical Center Greenville, NC United States; 2 Vidant Medical Center/East Carolina University Greenville, NC United States

**Keywords:** InHouse Call, communication, hospital directory, healthcare, health care, health informatics, mHealth, mobile app, digital health, patient records, electronic health

## Abstract

**Background:**

Communication failures disrupt physician workflow, lead to poor patient outcomes, and are associated with significant economic burden. To increase efficiency when contacting a team member in a hospital, we have designed an information directory app, InHouse Call.

**Objective:**

This study aimed to describe the design of InHouse Call, objectively compare the usefulness of the app versus that of traditional methods (operator or pocket cards, etc), and determine its subjective usefulness through user surveys and a net promoter score (NPS).

**Methods:**

This pilot study utilizing before-after trials was carried out at a tertiary academic hospital and involved 20 clinicians, including physiatrists, hospitalists, internal medicine and family medicine residents, and advanced practice providers/nurse practitioners/physician assistants. InHouse Call was designed to efficiently supply contact information to providers through a simple, user-friendly interface. The participants used InHouse Call in timed trials to contact a health care team member in the hospital via a telephone call. The effectiveness of InHouse Call in connecting the user with a contact in the hospital was measured through timed trials comparing the amount of time spent in attempting to make the connection using traditional methods versus the app. Usability was measured through exit surveys and NPS.

**Results:**

The average time spent connecting to the correct contact using traditional methods was 59.5 seconds, compared to 13.8 seconds when using InHouse Call. The degree of variance when using traditional methods was 1544.2, compared to 19.7 with InHouse Call. A call made using the traditional methods deviated from the mean by 39.3 seconds, compared to 4.4 seconds when using InHouse Call. InHouse Call achieved an NPS of 95.

**Conclusions:**

InHouse Call significantly reduced the average amount of time spent connecting with the correct contact as well as the variability to complete the task, thus proving to be the superior method of communication for health care providers. The app garnered a high NPS and positive subjective feedback.

## Introduction

### Background

The current fast-paced field of health care requires frequent communication among all health care team members including providers, case managers, nursing staff, therapists, pharmacists, nutritionists, technicians, and more. Owing to rapid expansion, hospitals in the United States often have their communications systems partitioned as opposed to a unified structure. Thus, communication failures have been a main source of concern in poor patient outcomes and often identified as a root cause of fatal errors [1]. In fact, entire simulation-based trainings have been dedicated to improving physician communication [2]. Although there have been several technological advancements in hospital communication in the past several decades, they have often acted as mere add-ons to an already complex communication system [3], and there has been limited evidence for improvement in effective interprofessional communication [4]. The time lost owing to poor hospital communication resulting in delayed testing and increased admission days comes at a significant economic burden with yearly estimates ranging US $12-$30 billion [5,6].

In large hospitals, there are two traditional methods used to contact a team member: (1) calling the operator and waiting to be connected and (2) pocket cards or workstation sheets with lists of general numbers. Many health care providers associate the operator method with long wait times and dropped or incorrectly transferred calls. While small and compact, pocket cards take up space in an already cluttered coat pocket, and their type font is often small and difficult to read. Both pocket cards and workstation sheets are physically limited in terms of the amount of information they can contain and sometimes have outdated information. Lamination of pocket cards prevents addition of information, while scribbling additional numbers onto the margins of workstation sheets leads to confusion.

Time-motion studies since the late 1980s have shown the downward trend of time spent in direct patient care and more time spent talking with physicians [7,8]. More recent studies have shown a sharper decline with the advent of the electronic health record system [9]. As health care becomes more complex, it is imperative to optimize and streamline time-consuming processes.

Technology is changing the face of health care. Physicians who adapt and work together with technology find success in a rapidly evolving system, and how well a physician adapts to a new technology is valuable to its success [10].

### Goals of This Intervention

To increase provider efficiency with contacting a team member in a large hospital, we designed and implemented a novel native information directory prototype app, InHouse Call. InHouse Call is a simple hospital directory tool used to facilitate seamless communication between providers and among health care team members by eliminating call transfers and making available the most up-to-date contact information for users. The user interface and back end of the app prioritizes ease of use and speed for completing the task.

### Research Goals

In this paper, we describe the design and future development of InHouse Call, objectively compare the usefulness of the app versus traditional methods via timed trials, and determine its subjective usefulness through user surveys and its net promoter score (NPS). The NPS is a standardized scoring system that rates the likelihood of a user recommending a new product or technology to a colleague [11].

## Methods

### Study Design and Setting

In this mixed methods study, we evaluated InHouse Call using a before-and-after survey methodology combined with objective timed trials. This study was performed at an academic medical center with a mix of 20 internal medicine and family medicine residents, hospitalists, and other inpatient clinicians.

### Intervention: InHouse Call

InHouse Call was developed by the author, a physical medicine and rehabilitation resident, using an iterative, user-centric interface with a focus on usability and efficiency [12]. The creation of the app was inspired by the difficulty experienced attempting to contact the hospital’s echocardiogram (ECHO) department while trying to complete a syncope work-up. This event resulted in an unnecessarily prolonged stay for a patient. Similar experiences shared by colleagues ultimately led to the development of InHouse Call.

**Figure 1:**
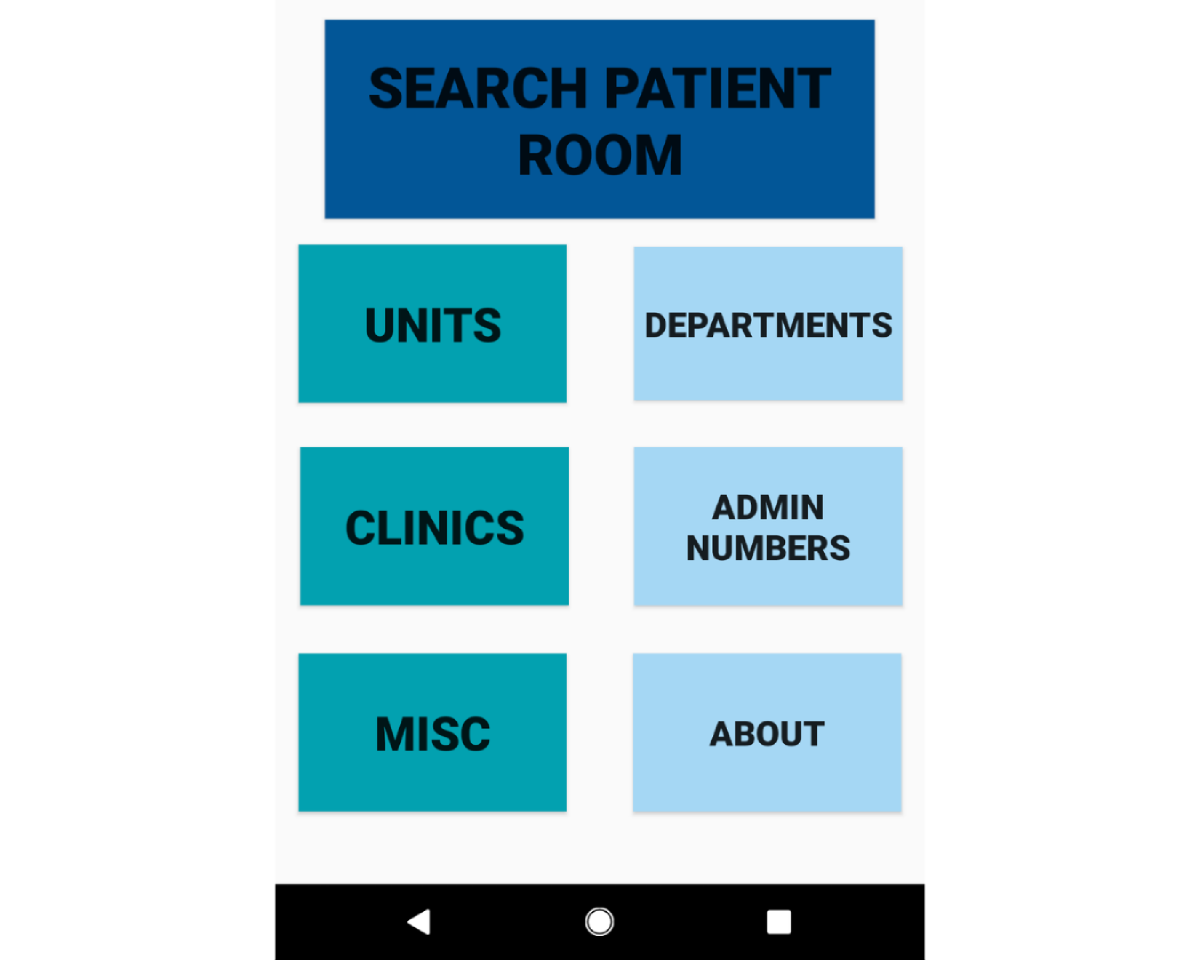
InHouse Call homepage with a searchable database and contact folders.

InHouse Call is a native mobile-based software app designed to supply the correct contact information to providers in the most efficient manner possible with a simple user-friendly interface (Figure 1).

InHouse Call is designed to contain the comprehensive database of health care team contact information found in large hospitals, including nursing staff, charge nurses, unit secretaries, case managers, pharmacists, nutritionists, and others, all searchable by the patient’s room number. The app also contains important department numbers, such as the radiology and laboratory departments, as well as hospital administration and clinic contact information. InHouse Call integrates seamlessly with the preexisting telephone system, as opposed to alternative health care messaging apps that work in a closed loop. It is important to note that InHouse Call is completely Health Insurance Portability and Accountability Act (HIPAA)–compliant as no personally identifiable or protected health information of any party is stored either locally or remotely.

In our app design, the user opens the app leading directly to a home screen providing the main “Search Patient Room” searchable database as well as six subfolders containing pertinent, easy-to-read information, including the following: Units, Departments, Clinics, Admin Numbers, Misc, and About. Unique to the InHouse Call design, the “Search Patient Room” database allows the user to enter the patient room number without requiring patient-identifiable information, which then provides fixed health care team member information assigned to that specific patient room. This includes the registered nurse (RN) pod or hallway phone, case manager, or pharmacist who is directly assigned to that room. It is important to note that the team member’s personal identifiable information is not included; only the phone number assigned to that team member is provided, thus ensuring HIPAA compliance.

**Figure 2:**
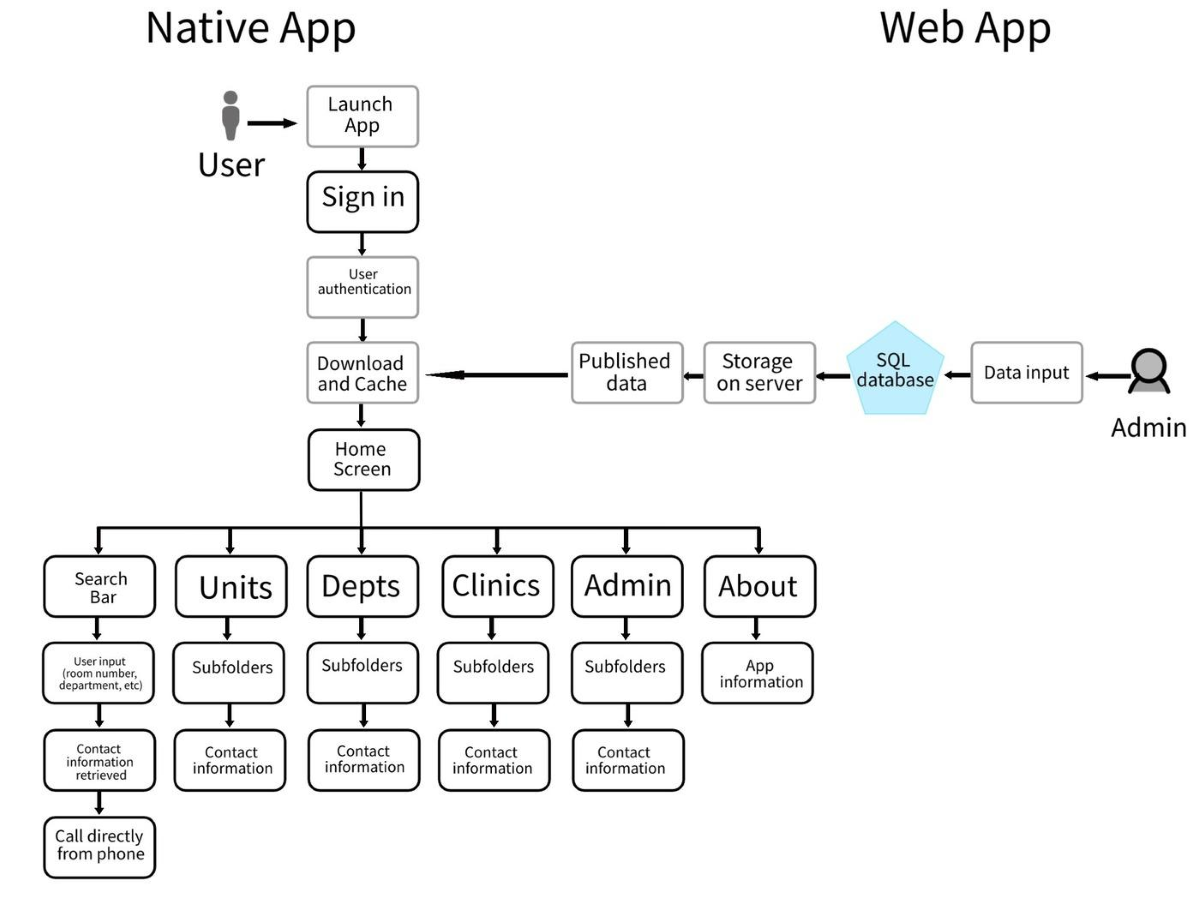
InHouse Call sitemap. Anticipated integration of a web app data input system to keep InHouse Call updated.

The app is currently in the native form and created with Android Studio and written in Java. Only textual data—no images or video—are stored on-premise in a SQLite database built into the app, using a dual-encrypted secure server. This design was chosen to ensure the highest-speed search output available in large hospital center environments where internet connectivity may not be 100% reliable, especially in corridors or elevators where physicians are often located while travelling between patient rooms. Although the current system is in a native app, Figure 2 describes the web app phase where the data will be stored on a server but still cached on the native app to ensure the same level of speed.

In the anticipated archistructure described in Figure 2, there are several noteworthy integration points for the future planned software. First, the user login and authentication process serve two purposes: (1) it provides security for the hospital contact information available, and (2) it allows a single provider to switch between different hospital directories. Clinicians often work at different hospitals, sometimes even between different health care systems, and this feature will allow them to log out of one hospital directory and log in to another hospital directory. Next, the download or cache step ensures that the most up-to-date information is available in the directory. This step is designed to balance speed with functionality. A key anticipated feature also includes calls being directly made from the user’s smartphone containing the app.

### Participant Selection

In total, 20 providers were surveyed and participated in the timed trials. The participants mainly included hospitalists and internal medicine and family medicine residents as they were found to require the most communication with health care team members. The study also included a large variety of clinicians who mainly work in the hospital setting, including surgery residents and nurse practitioners (NPs) or physician assistants (PAs), intensivists, pediatricians, endocrinologists, physiatrists, and emergency medicine and obstetrics and gynecology residents. This was done to test the usefulness of the app by comparing providers who had high and low call volumes.

### Study Protocol

Before the introduction of InHouse Call, the participants completed a baseline survey of their experience with the current hospital communication system, including its impact on their workflow and how much time they spent searching for the correct contacts each day. All participants completed the baseline survey. After the completion of the survey, the clinicians then participated in 2 rounds of timed trials, one using their preferred traditional methods of either calling the operator or using a pocket card/workstation contact list, and one using InHouse Call.

For the first round of the timed trial, a piece of paper was placed face down on the table in front of the participant at their workstation, and the participant was instructed to complete the task outlined on the piece of paper; namely, to make a phone call either to a case manager, nurse, department, etc. The participant was told that the timer would start when the piece of paper was turned over and end when the call to the correct contact rang once. The contacts were made aware ahead of time that their phones may be ringing temporarily.

Before the second round of the timed trial, the participants were then briefly introduced to InHouse Call. They were given 45-60 seconds to become familiar with its functionality. They were shown how to locate a contact number using the “Search By Patient Room” feature as well as by the department and clinic subfolders. The same trials were conducted again with a smartphone placed on the workstation table with the screen off and app closed. These providers were instructed to again follow the prompts on the piece of paper in front of them, this time using InHouse Call to complete the task. In total, 85% of the participants completed the trial phase of the study. The providers who declined to participate in the timed trials were still introduced to InHouse Call and its functionality.

The final phase of the study was an exit survey. Although only 85% of the participants completed the trial phase of the study, 100% of participants were shown and interacted with InHouse Call and completed the exit survey, which included the NPS questionnaire. Survey respondents were also asked about their comfort level with the app to determine its usability.

### Outcome Measures

We evaluated InHouse Call’s effectiveness in connecting the user with the correct contact in the hospital through the use of objective timed trials and comparing the amount of time spent in attempting to make the connection using traditional methods (operator or pocket cards, etc) versus InHouse Call. Usability was further quantified through the use of the NPS and the exit survey.

### Analysis Approach

We classified the participants by their area of practice, their number of calls made in a workday, and their experience with the current communication system. We then compared the results of the timed trials using the traditional communication methods versus InHouse Call. The results of the two methods were further compared by average time, SD, and variance. The NPS was obtained to determine the successful rollout of the app, and the feedback obtained in the exit survey was categorized into general themes or categories.

Qualitative data were managed and analyzed using Google Sheets (Alphabet Inc).

## Results

### Characteristics of Study Subjects

There was a 100% response rate in the entrance and exit survey for the 20 participants, while 85% of participants completed some or all of the timed trials. Participants were classified in accordance with the area of practice and number of calls made in an average work day (Tables 1 and 2).

**Table 1:** Composition of the participating health care providers.

Provider specialty	Proportion, %
Obstetrics and gynecology	5
Emergency medicine	5
Pediatrics	5
Neonatal intensive care unit nurse practitioner	5
Endocrinology	5
Physical medicine and rehabilitation	10
Surgery	20
Internal medicine, family medicine, or hospitalist	45

**Table 2:** Average number of calls made per participant.

Calls, n	Participants, n (%)
0-5	1 (5)
6-10	7 (35)
11-15	5 (25)
16-20	4 (20)
21-25	1 (5)
26-30	2 (10)

### Survey Results

In the entrance survey, 55% of respondents reported that it took over a minute to connect with a health care team member through the operator, while 65% reported the frequency of dropped calls or being transferred to the wrong person when using the operator as either occasionally or frequently (Tables 3 and 4).

In total, 75% of respondents reported frustration over not being able to find the right contact daily or several times per week, and 70% reported that poor communication impacted patient care and workflow daily or several times per week (Table 5). None responded that they were never frustrated by the inability to contact the right person, or that poor communication never impacted patient care and workflow.

A total of 80% of respondents reported spending at least 5 minutes of their workday searching for the right contact, with 10% reporting spending at least 20 minutes of their workday (Table 6). When asked to determine the number 1 complaint with communication in their hospital, 55% of respondents reported “Hard to find the right number” (Table 7).

**Table 3:** Perceived time spent with the operator to determine how long respondents felt it would take them to connect with a health care team member (registered nurse, case manager, etc) through the switchboard.

Self-reported perceived time (minutes)	Participants (n=16)^a^, n (%)
<0.5	0 (0)
0.5-1	3 (15)
<1	2 (10)
1-1.5	6 (30)
1.5-2	3 (15)
2.2-5	0 (0)
>2.5	2 (10)

^a^Drop-out rate=20% (n=4 participants).

**Table 4:** Frequency of wrong transfers or dropped calls among respondents when using the switchboard method to reach a health care provider.

Frequency	Respondents (n=18)^a^, n (%)
Never	0 (0)
Very rarely	2 (10)
Rarely	3 (15)
Occasionally	6 (30)
Frequently	7 (35)
Always	0 (0)

^a^Drop-out rate=10% (n=2 participants).

**Table 5:** Frequency of frustration among respondents (N=20) on not being able to find the right contact and poor communication affecting patient care and workflow.

Frequency	Respondents frustrated on not finding the right contact, n (%)	Poor communication affecting care delivery and workflow among respondents, n (%)
Never	0 (0)	0 (0)
Once per month	0 (0)	1 (5)
Several times per month	2 (10)	4 (20)
Once per week	3 (15)	1 (5)
Several times per week	8 (40)	8 (40)
Daily	7 (35)	6 (30)

**Table 6:** Time spent by respondents (N=20) in searching the right contact on each day.

Time (minutes)	Respondents, n (%)
0-5	4 (20)
5-10	7 (35)
10-20	7 (35)
20-30	1 (5)
30-60	1 (5)
>60	0 (0)

**Table 7:** Primary complaint of respondents (N=20) with communication at the hospital.

Complaint	Respondents, n (%)
Transferred to the wrong person	1 (5)
Takes too much time	5 (25)
Difficult to find the right number	11 (55)
All three circled	1 (5)
Unable to reach intended person	1 (5)
Poor interdepartmental communication	1 (5)

### Timed Trials Results

Of the 20 survey respondents, 75% participated in the timed trial to reach an RN. The average participant spent 78.7 seconds to reach an RN via traditional methods and spent only 16 seconds when using InHouse Call (Table 8). Of the 15 trials using traditional methods, one participant (participant 8) gave up after 2 minutes owing to incomplete information available on their pocket card, while another (participant 12) spent 203 seconds connecting to the right contact after experiencing a dropped call.

Of the 20 survey respondents, 60% participated in the timed trial to reach the ECHO department. The average participant spent 49.9 seconds to reach the ECHO department via traditional methods and spent 11.1 seconds via InHouse Call (Table 8). Of the 12 trials using traditional methods, 3 participants gave up (participants 6, 10, and 11): one owing to incomplete information available on their pocket card, one owing to long wait times, and the last one owing to a wrong transfer.

Of the 20 survey respondents, 11 (55%) participated in the timed trial to reach a clinic. The average participant spent 43.9 seconds to reach a clinic via traditional methods and spent 13.8 seconds via InHouse Call (Table 8). Of the 11 trials using traditional methods, 2 participants (participants 6 and 10) gave up owing to incomplete information available on their pocket card.

Of the 20 survey respondents, 6 (30%) participated in the timed trial to reach a wound care team member. All participants were unable to complete the task using traditional methods, averaging 161.8 seconds before giving up and averaging 19.2 seconds via InHouse Call with success (Table 8). The longest time a participant attempted to reach a wound care team member using traditional methods was a little over 300 seconds.

Out of 44 trials in total, the average time spent to connect to the correct contact, via traditional methods was 73.5 seconds, while the average time spent to connect to the correct contact via InHouse Call was 14.6 seconds (Table 9). As no participant was able to connect with a wound care team member via traditional methods, that set of data collected may be considered outliers. With the wound care team outlier data removed, the average time spent to connect to the correct contact via traditional methods was 59.5 seconds while the average time spent via InHouse Call was 13.8 seconds.

Also shown in Table 9 is the degree of variability between each call using traditional methods versus the consistency achieved using InHouse Call. Analyzing the data without outliers, the degree of variance using traditional methods was 1544.2 compared to 19.7 with InHouse Call. A call made using the traditional methods deviated from the mean by 39.3 seconds, while a call using InHouse Call deviated from the mean by 4.4 seconds.

**Table 8:** Time to reach different health care providers by using the traditional method versus InHouse Call.

Participant	Time to reach registered nurses (seconds)^a^		Time to reach the echocardiogram department (seconds)^b^		Time to reach a clinic (seconds)^c^		Time to reach the wound care team (seconds)^d^	
	Traditional method	InHouse Call	Traditional method	InHouse Call	Traditional method	InHouse Call	Traditional method	InHouse Call
1	100	20	18	10	24	15	62	20
2	48	11	70	14	58	25	121	30
3	55	17	52	10	62	15	123	12
4	33	20	50	19	45	15	180	22
5	37	17	15	5	30	10	183	16
6	25	20	63	12	61	12	302	15
7	85	10	100	10	51	14	N/A^e^	N/A
8	127	14	20	12	15	15	N/A	N/A
9	68	20	41	10	25	10	N/A	N/A
10	47	12	120	13	60	13	N/A	N/A
11	58	12	17	11	52	8	N/A	N/A
12	203	16	33	7	N/A	N/A	N/A	N/A
13	124	20	N/A	N/A	N/A	N/A	N/A	N/A
14	43	10	N/A	N/A	N/A	N/A	N/A	N/A
15	128	22	N/A	N/A	N/A	N/A	N/A	N/A

^a^Two participants gave up on the task on using traditional methods.

^b^Three participants gave up on the task on using traditional methods.

^c^Two participants gave up on the task on using traditional methods.

^d^All 6 participants gave up on the task on using traditional methods.

^e^N/A: not applicable.

**Table 9:** Comparison of timed trials by average time, SD, and variance.

	Average time	Mean deviation	Variance (SD)	*t* test (*df*)
Traditional methods	73.50	42.75	3328.52 (57.69)	<0.001 (43)
InHouse Call	14.57	3.98	25.02 (5.00)	N/A^a^
Traditional methods without wound team data	59.55	28.28	1544.19 (39.30)	<0.001 (4)
InHouse Call without wound team data	13.84	3.62	19.71 (4.44)	N/A

^a^N/A: not applicable.

### NPS

All participants completed the exit survey after taking the initial survey and being introduced to InHouse Call (Table 10). Of the 20 respondents to the exit survey, 95% (19/20) scored the likelihood of recommending InHouse Call to a friend as a ≥9 on a 10-point Likert scale, and were therefore classified as promoters. In total, 5% of the respondents scored this same question as 7 or 8 and were classified as neutral, while none provided a score of ≤7. This yielded an NPS of 95.

**Table 10:** Exit survey results.

Likeliness/usefulness rating^a^	Respondents’ answers (N=20), n (%)			
	How useful did you find InHouse Call?	How comfortable were you using InHouse Call?	How likely would you use InHouse Call in your daily work?	How likely would you recommend InHouse Call to another coworker?
1-7	0 (0)	0 (0)	0 (0)	0 (0)
8	1 (5)	2 (10)	3 (15)	1 (5)
9	2 (10)	4 (20)	1 (5)	2 (10)
10	17 (85)	14 (70)	16 (80)	17 (85)

^a^1=least likely, 10=most likely.

### Feedback Results

Nearly all survey respondents participated in the optional write-in feedback section of the exit survey (Table 11). Participants identified three major categories of feedback on InHouse Call: (1) ease of use, (2) efficiency and usefulness in daily work, and (3) opportunities for improvement. Table 11 summarizes these categories and provides participant quotes for illustrative purposes.

Half of the participants commented on the ease of use, with some describing the interface as “user-friendly” and “intuitive.” Nearly half of participants commented on the app’s efficiency, with several mentioning the benefit of not having to wait on hold while making a telephone call.

**Table 11:** Exit survey with quotes.

Category of feedback			Example quotes
Ease of use			“Love the easy access to all necessary #'s, esp RN pods.” [PGY-4 Endocrinology Fellow] “Awesome, easy to use, time saver, eliminates hassle of searching numbers.” [Trauma Surgery advanced practice provider/nurse practitioner/physician assistant]
Efficiency and usefulness in daily work			“App would be very useful.” [Hospitalist] “That is so much easier than using pocket cards or calling a main number to try to reach another department. This app would greatly improve my productivity.” [PGY-3 Internal Medicine]
**Opportunities for improvement**			
	Can provide even further available information, such as other departments and clinics	“I would add charge nurse info in the room assignment search result. Make sure things like GI lab + pulm lab, etc.” [General Surgery advanced practice provider/nurse practitioner/physician assistant]	
	Can add other information such as on call services and updated admission algorithms	“ICU Attending #, VIR, CT Surgery, Off site surgeons (example: southern surgical)...agree with algorithm admissions, consult services.” [PGY-3 Emergency Medicine]	

## Discussion

### Principal Findings

In a series of trials with a variety of providers, mostly comprising internal medicine and family medicine residents and hospitalists but including surgical NPs or PAs and subspecialists, a total of 88 timed telephone calls were conducted as part of the timed trials to assess the effectiveness of InHouse Call. Traditional methods, such as using the operator services or pocket cards are cumbersome, antiquated methods for making calls in the hospital setting, and resulted in average trial times of 73.5 seconds per call.

By eliminating these time-consuming steps, the average time saved by using InHouse Call ranged from 45.71 seconds to 58.93 seconds. The time saved is significant when added over longer periods of time and with larger pools of hospital clinician users. Even when considering only the 10% of survey respondents who self-reported making 26-30 calls per day, the app would save those clinicians approximately 30 minutes of time spent on the phone per work day. This could be extrapolated by the estimated amount of phone calls made from providers to the hospital operator of 1000 calls per day, and we begin to see over 5000 work hours saved per year.

Wait times, transfers, and dropped calls were a major factor in the large degree of variance when using traditional methods to make a call. Conversely, InHouse Call eliminated these variable factors and streamlined the process, resulting in a more consistent outcome. This standardization in the process demonstrates the app’s efficiency and was also reflected in the participants’ feedback.

InHouse Call received an overwhelmingly positive response from its users with a strong NPS of 95, owing largely in part to the way it directly addressed participants’ top complaints with the hospital communication system of “taking too much time” and “difficulty finding the right number.” Eliminating long hold times and call transfers addressed the time-consuming complaint, while the “Search Patient Room” database and subfolders addressed the difficulty in finding the correct contact. InHouse Call uses the patient’s room number as an invariant through which a large amount of contact information can be quickly accessed. The subfolders are also effective at organizing contact information in large departments such as the radiology department with several modalities such as computed tomographic scan, magnetic resonance imaging (MRI), ultrasound, and ECHO available. The subfolders quickly and efficiently guide the user to the correct contact information compared to when using an often difficult to read pocket card.

Although the tallies for average time saved and an NPS of 95 are good prognostic indicators for the potential reliability of InHouse Call in a clinician’s daily work, there are several future features which will further improve the app’s utilization. Chief among them is the recently added ability to make calls directly from the app, thus further streamlining the process by eliminating the need to manually dial the number into the clinician’s workstation phone. Furthermore, making the contact information in the subfolders (MRI, clinics, etc) part of the searchable database would further streamline the process of accessing that information. Survey respondents provided feedback for possible additional features including on-call providers and an admission algorithm, which may eliminate calls to the incorrect admission team.

In the exit survey, the lowest-scoring question was “How comfortable were you using InHouse Call?” despite feedback from the same survey respondents indicating that there was significant ease of use and an intuitive user interface. This may be accounted for by the fact that the respondents only had 45-60 seconds to familiarize themselves with the app before beginning their trials and filling out the exit survey. Their ease and comfort might be higher with a longer exposure time to the app and its functionality. A larger pilot study of tracking the app’s usage in the clinician’s workday would be advantageous to more accurately determine the app’s integration into a provider’s workflow. A system usability scale (SUS) would also be beneficial to quantifiably ascertain InHouse Call’s usability in addition to the exit survey and feedback provided.

### Comparison With Preexisting Systems

Improved hospital communication has been a focus of modern innovation for the past several decades. Several large medical centers have relied on direct messaging systems such as Cureatr, Cortext, and Voalte [13]. Although adequate in solving the problem of communication, the major limitation with these systems is that the name of the health care team member contact is required to initiate the communication; nonetheless, the team members’ shifts change too frequently for this information to be kept up to date [14]. Further, these direct messaging systems only communicate with each other in a closed loop and thus add to an already complex hospital communication system. InHouse Call addresses these shortcomings by first having a database that is centered around a patient's room, and second by utilizing the already existing hospital telephone system with which users are familiar.

Some attempts have been made to create comprehensive hospital directories, but these systems have not reached mainstream success [15]. Their limitations include having their contact information crowd-sourced [16], or their systems were unable to alleviate frustration over finding the appropriate contact [17]. Studies regarding the usability and impact of other communication systems on clinical practice are limited; however, to our knowledge, this is the first study comparing timed trials of actual providers in their natural work environment.

### Limitations

There are some limitations to this study. Since the app developer and author was the person who conducted the study, there is room for observation biases in the timed trials. However, the effectiveness of the app is self-evident in the exceptional time-saving results achieved. Further, the independent user feedback obtained in the exit strategy praised the app’s time-saving functionality. Participant bias is also a risk owing to the participants being able to inherently see the aim of the study and thus put forth varying effort during the trials. Attempts to mitigate this bias were made by limiting the exposure of the participant to InHouse Call before the completion of the first round of the timed trials. In addition, the absence of incentives and rewards for the participants help to decrease bias with the results. Since the author and facilitator is also the app developer, there is an inherent conflict of interest not unique to studies of novel innovations. Conducting a larger study with more degrees of separation between the developer and participants would help mitigate this. Another limitation of the study was that it was performed at a single hospital. Although utilizing a phone operator system is common in all hospitals, there may be variables in different phone networks, which may impair InHouse Call’s usability across all health care systems. However, InHouse Call is designed with the fundamental structure of a hospital in mind by utilizing the patient room number as the keystone of the contact database. Finally, owing to the busy work schedules of the participants and the somewhat lengthy study design, the number of volunteers, though diverse, was limited. Thus, the sample size was small. A larger cohort study would provide a more accurate insight into the app’s receptivity.

### Conclusions

We designed and implemented a novel native information directory app, InHouse Call, and found that its application to the average provider’s workday saves a significant amount of time in placing calls. By eliminating wait times, call transfers, and dropped calls, the average amount of time to initiate and complete a call was significantly reduced as well as the variability of time to complete the task. Users found the app easy to use, effective, and useful for their daily work. The NPS was an astounding 95, which is on par with other great apps. Despite its current effectiveness, opportunities for improvement were also determined. As InHouse Call relies on the current telephone system already universally found in large hospitals, it has the potential to be expanded to nearly all other institutions.

## Abbreviations

APP: advanced practice provider/nurse practitioner/physician assistant
ECHO: echocardiogram
HIPAA: Health Insurance Portability and Accountability Act
MRI: magnetic resonance imaging
NP: nurse practitioner
NPS: net promoter score
PA: physician assistant
RN: registered nurse
